# Validation of a semi-automated method to quantify lesion volume changes in multiple sclerosis on 2D proton-density-weighted scans based on image subtraction

**DOI:** 10.1016/j.ynirp.2023.100194

**Published:** 2023-12-21

**Authors:** Rozemarijn M. Mattiesing, Serena Stel, Alysha S. Mangroe, Iman Brouwer, Adriaan Versteeg, Ronald A. van Schijndel, Bernard M.J. Uitdehaag, Frederik Barkhof, Hugo Vrenken, Joost P.A. Kuijer

**Affiliations:** a MS Center Amsterdam, Radiology and Nuclear Medicine, Amsterdam Neuroscience, Amsterdam UMC location VUmc, De Boelelaan 1118, 1081 HZ, Amsterdam, the Netherlands; b MS Center Amsterdam, Neurology, Amsterdam Neuroscience, Amsterdam UMC location VUmc, De Boelelaan 1118, 1081 HZ, Amsterdam, the Netherlands; c Queen Square Institute of Neurology and Centre for Medical Image Computing, University College London, London, UK

**Keywords:** Image subtraction, Magnetic resonance imaging, Semi-automated segmentation, White matter lesions, Lesion volume changes, Multiple sclerosis

## Abstract

**Background:**

The detection and quantification of changes in white matter lesions in the brain is important to monitor treatment effects in patients with multiple sclerosis (MS). Existing automatic tools predominantly require FLAIR images as input which are not always available, or only focus on new/enlarging activity. Therefore, we developed and validated a semi-automated method to quantify lesion volume changes based on 2D proton-density (PD)-weighted images and image subtraction. This semi-automated method provides insight in both “positive” activity (defined as new and enlarging lesions) and “negative” activity (disappearing and shrinking lesions).

**Methods:**

Yearly MRI scans of patients with early MS from the REFLEX/REFLEXION studies were used. The maximum follow-up period was 5 years. Two PD-weighted images were normalized, registered to a common halfway-space, intensity-matched, and subsequently subtracted. Within manual lesion masks, lesion changes were quantified using a subtraction intensity threshold and total lesion volume change (TLVC) was calculated. Reproducibility was measured by assessing transitivity, specifically, we calculated the intraclass correlation coefficient for the absolute agreement (ICC_trans_) and the difference (Δ_trans_) between the direct one-step and indirect multi-step measurements of TLVC between two visits. Accuracy was assessed by calculating both the intraclass correlation coefficient for absolute agreement (ICC_acc_) and the difference (Δ_acc_) between the one-step semi-automated TLVC and manually measured lesion volume change (numerical difference) between two visits. Spearman's correlations (r_s_) were used to assess the relation of global and central atrophy, manually measured T2 lesion volume, and lesion volume change with the method's performance as reflected by the difference measures |Δ_trans_| and Δ_acc_. An alpha of 0.05 was used as the cut-off for significance.

**Results:**

Reproducibility was excellent, with ICC_trans_ values ranging from 0.90 to 0.96. Accuracy was good overall, with ICC_acc_ values ranging from 0.67 to 0.86. The standard deviation of Δ_trans_ ranged from 0.25 to 0.86 mL. The mean of Δ_acc_ ranged from 0.11 to 0.37 mL and was significantly different from zero. Both global and central atrophy significantly correlated with lower reproducibility (correlation of |Δ_trans_| with global atrophy, r_s_ = −0.19 to −0.28, and correlation of |Δ_trans_| with central atrophy, r_s_ = 0.22 to 0.34). There was generally no significant correlation between global/central atrophy and accuracy. Higher lesion volume was significantly correlated with lower reproducibility (r_s_ = 0.62). Higher lesion volume change was significantly correlated with lower reproducibility (r_s_ = 0.22) and lower accuracy (correlation of Δ_acc_ with lesion volume change, r_s_ = −0.52).

**Discussion:**

The semi-automated method to quantify lesion volume changes has excellent reproducibility and overall good accuracy. The amount of atrophy and especially lesion volume (change) should be taken into account when applying this method, as an increase in these variables might affect the quality of the results.

**Conclusion:**

Overall, the semi-automated subtraction method allows a valid and reliable quantitative investigation of lesion volume changes over time in (early) MS for follow-up periods up to 5 years.

## Highlights

• We developed and validated a semi-automated method to quantify lesion volume changes.
• The method has excellent reproducibility and overall good accuracy.
• Increasing atrophy and lesion volume (change) have a limited impact on performance.
• The method reliably quantifies lesion volume changes in multiple sclerosis.

## 1 Introduction

In multiple sclerosis (MS), magnetic resonance imaging (MRI) is widely used to detect and monitor the evolution of white matter (WM) abnormalities (lesions) in the brain. More specifically, T2 lesions are part of the MRI features that are commonly assessed. T2 lesions appear hyperintense on proton-density (PD)-/T2-weighted images, and on fluid-attenuated inversion recovery (FLAIR) images. The total volume of T2 lesions is used in clinical trials to evaluate the effects of disease modifying treatments on disease activity.

To quantify changes in lesion volume over time, a series of images has to be segmented. Performing this manually requires a lot of expertise and is a labor-intensive process. Therefore, automated change detection methods such as the lesion segmentation tool (Schmidt et al., 2019) have been developed. Existing tools predominantly require FLAIR images. However, these images are not always available in clinical practice and in many trials in which dual-echo PD/T2 images are commonly acquired. For this reason, we developed a semi-automated lesion change quantification method that is based on 2D PD-/T2-weighted images and image subtraction.

Subtraction images are obtained by subtracting two registered serial MRI scans after image intensity matching. This cancels out stable non-active lesions, which leads to an enhanced contrast between active lesions and the background (Moraal et al., 2009, 2010) allowing detection of changes in lesion load and quantifying positive and negative disease activity.

Few automated methods based on image subtraction exist (e.g., Battaglini et al., 2014; Ganiler et al., 2014). However, these methods focus on lesion numbers and/or new/enlarging lesions only. The currently proposed method also localizes and quantifies disappearing and shrinking lesions.

The aim of the current study was to validate a semi-automated lesion change quantification method for a one-year time interval between MR imaging. The performance was assessed by quantifying the reproducibility and accuracy of the proposed method when using shorter periods between serial MRI scans. The limits of the method were also tested, by investigating its performance when applied to longer intervals (up to 5 years). Finally, the potential influence of brain atrophy and lesion volume (change) on the reproducibility and accuracy were investigated.

## 2 Methods

### 2.1 Description of the study and dataset

Five-yearly imaging data from the REFLEX/REFLEXION (REbif FLEXible dosing in early MS/extensION; NCT00404352/NCT00813709) studies were used. REFLEXION was a preplanned extension of the REFLEX study to evaluate the effects of early and delayed treatment with subcutaneous interferon beta-1a in patients with early MS over a long-term follow-up period (Comi et al., 2017). Table 1 provides an overview of the different time intervals that were investigated in the current study and the corresponding demographics of the included patients.

**Table 1:** Demographics of the included patients for each interval.

Years	Interval	Number of patients	Age, years (range, mean ± SD)	Female, n (%)
1	BSLN-M12	328	17-51 (31.60 ± 8.30)	210 (64%)
1	M12-M24	338	17-51 (31.66 ± 8.32)	214 (63.3%)
1	M24-M36	290	17-51 (31.60 ± 8.43)	177 (61%)
1	M36-M48	281	17-51 (31.78 ± 8.47)	170 (60.5%)
1	M48-M60	275	17-51 (31.85 ± 8.44)	164 (59.6%)
2	M36-M60	235	17-51 (31.88 ± 8.48)	136 (57.9%)
3	M24-M60	220	17-51 (31.70 ± 8.26)	128 (58.2%)
4	M12-M60	207	17-51 (31.94 ± 8.27)	122 (58.9%)
5	BSLN-M60	196	17-51 (32.06 ± 8.25)	118 (60.2%)

BSLN = baseline, M = month, SD = standard deviation.

All study sites (N = 70) were required to follow an MRI acquisition protocol that specified a preference for 1.5 T scanners. The yearly MRI scans consisted of 1 × 1 × 3 mm^3^ axial 2D dual-echo PD-/T2- (which means both sequences were generated in a single acquisition; TR, 2000–3000 ms; TE1/TE2, 20–30 ms/80–100 ms), and T1-weighted (TR, 400–600 ms; TE, 10–16 ms) spin echo images with full brain coverage (number of slices, 46). Manual delineations of the lesions on the PD-weighed images and manually edited brain extraction masks originally obtained by using the FMRIB software library (FSL) (Smith et al., 2004) brain extraction tool (Smith, 2002) with the T1-weighted image as input, were previously created in the context of the REFLEX/REFLEXION studies by the Image Analysis Center of Amsterdam UMC (Location VUmc, Amsterdam, the Netherlands).

### Ethics approval

This study used data from the REFLEX and REFLEXION studies, which were undertaken in compliance with the Declaration of Helsinki and standards of Good Clinical Practice according to the International Conference on Harmonization of Technical Requirements for Registration of Pharmaceuticals for Human Use. Before initiation of the studies at each center, the relevant institutional review board or independent ethics committee reviewed and approved the study protocols, patient information leaflets, informed consent forms, and investigator brochures. All patients provided written informed consent at the screening visit of REFLEX, and before enrollment to REFLEXION.

### 2.2 Description of the semi-automated method

#### 2.2.1 Brief overview

**Fig. 1:**
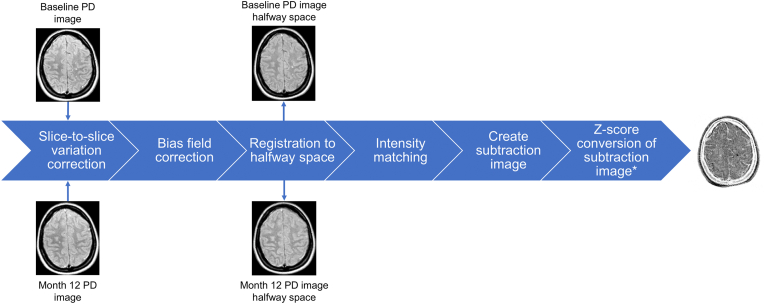
Schematic depiction of the processing pipeline of the semi-automated method. *based on mean and standard deviation within brain tissue mask excluding lesions, PD = proton-density.

**Fig. 2:**
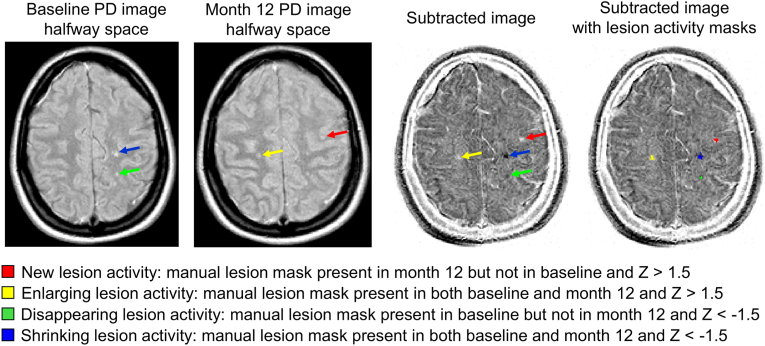
Quantification of different categories of lesion change based on manual lesion masks and the subtraction image converted in Z-scores. PD = proton-density.

Fig. 1, Fig. 2 summarize the analysis pipeline. Briefly, substantial differences in signal intensity between odd and even slices of the PD-weighted images were corrected for each visit. Then, after bias field correction, the PD-weighted images were registered to a common halfway space. The intensity distributions of the two halfway PD images were matched and subsequently these images were subtracted. The subtraction image intensities were transformed into Z-scores. Based on the manually created lesion masks as a reference and a threshold of |Z| > 1.5, the voxels inside the manual lesion masks were classified according to different categories of lesion change. The total lesion volume change was calculated by subtracting the negative activity (disappearing + shrinking voxels) from the positive activity (new + enlarging voxels).

#### 2.2.2 Pre-processing and creation of subtraction images

The processing pipeline as illustrated in Fig. 1 was implemented using several tools of FSL (version 5.0.6). As the T1- and PD-/T2-weighted images were acquired using different sequences, registration was necessary. For this purpose, the registration of the native T1-weighted image to the native T2-weighted image was first calculated. T2-weighted images were chosen because these provide better contrast between cerebrospinal fluid and brain tissue compared to the PD-weighted images (which provide better contrast between cerebrospinal fluid and lesions) that were generated in the same dual-echo acquisition, which is beneficial to the registration accuracy. The registration of the T1-weighted image to the T2-weighted image was calculated using FSL-FLIRT (Jenkinson et al., 2002). A normalized mutual information cost function, suitable for images with different types of tissue contrast, was used. As these two different sequences originate from the same subject within the same session, rigid body registration (6 degrees of freedom) was optimal. Then, the obtained transformation was applied with nearest neighbor interpolation to bring the existing T1-weighted brain mask to the T2-weighted image matrix. This resulted in a PD-/T2-weighted brain mask. Next, the PD-weighted images were pre-processed. The first preprocessing step consisted of removal of signal intensity differences between the odd and even slices in the PD-weighted images. Such difference may arise from an interleaved slice acquisition order. This intensity correction was performed by matching, within the PD-/T2-weighted brain mask, the average signal of the odd slices and of the even slices. Subsequently, the tool fsl_anat (https://fsl.fmrib.ox.ac.uk/fsl/fslwiki/fsl_anat) was used for bias field correction. FSL-FLIRT was used with default options (correlation ratio cost function and 12 degrees of freedom), T2 images of both visits as input, and brain masks as weights, to calculate the registration between both visits in both directions. These two transforms were used to calculate a halfway space following the procedures applied in FSL-SIENA (Smith et al., 2001, 2002), except that in this case the registration is based on the brain images alone without using the skull. Then the PD- and T2-weighted images, PD-/T2-weighted brain masks, and lesion masks from two time-points were treated in a symmetrical way by registering these to the common halfway space using sinc (images), nearest neighbor (brain masks), and trilinear (lesion masks) interpolation. In the next step, the signal intensity distributions of the two PD halfway images were matched. This matching of histograms was achieved by the following steps. First, the intensity range of each PD halfway image was normalized to the interval 0–1 by linear scaling. Then the T2 halfway images were brain masked and the resulting image was segmented into two classes (brain and cerebrospinal fluid) by running FSL-FAST (Zhang et al., 2001). To prevent exclusions of lesions from brain tissue, the halfway-registered lesion masks were added to the brain tissue class. The corresponding PD halfway image was masked by this “brain tissue + lesions” mask and then the itkHistogramFilter tool was used on the resulting PD halfway images to determine the required histogram changes. These histogram changes were then applied to the full halfway PD images (i.e., including the skull, meninges etc.) by using the itkHistogramFilter tool again. Finally, to create the subtraction image, the image of the first visit was subtracted from that of the second visit.

#### 2.2.3 Quantification of lesion change

To quantify the lesion volume change, the following steps were applied. In order to account for the differences between sites and scanners, the subtraction image intensities were converted into Z-scores based on the mean and standard deviation (SD) within the brain tissue mask (as described in the previous section) excluding the lesions. The resulting Z-score maps were used to classify the activity of all voxels in categories “positive”, “negative”, or “none”. This was performed by applying a uniform threshold of Z > 1.5 for positive activity and Z < −1.5 for negative activity, which was determined through heuristic optimization on a subset of cases. To refine this classification based on the Z-scores, the change analysis was restricted to the manual lesion masks in order to avoid false positive lesion change measurements on the subtraction images due to noise or image artifacts. Trilinear interpolation was used to register these manual lesion masks from both visits to the halfway space and then to ensure the inclusion of lesion boundaries a relatively low threshold of 0.25 was applied. Isolated single voxels in an image slice were removed.

Three different situations were then distinguished based on the presence or absence of a manual lesion mask at the two visits, for each individual lesion. 1) In case of a newly appearing lesion on visit 2, the manual lesion mask for that lesion did not overlap with any manual lesion mask from visit 1, and the voxels with Z > 1.5 within this manual lesion mask were defined as “new activity”. 2) In the case of a disappearing lesion that was present on visit 1, a manual lesion mask for that lesion did not overlap with any manual lesion mask from visit 2, and the voxels with Z < −1.5 within this manual lesion mask were defined as “disappearing activity”. 3) In the case of a changing lesion that was present at both visit 1 and 2, the manual lesion masks from both visits (partially) overlapped. Voxels within this “combined” manual lesion mask with an intensity Z > 1.5 were defined as “enlarging activity” and Z < −1.5 as “shrinking activity”. An additional restriction was that enlarging or shrinking activity could not occur in the eroded manual lesion mask of visit 1 or visit 2, respectively. The total lesion volume change (TLVC) was calculated by subtracting the volume of the voxels categorized as negative lesion activity (disappearing and shrinking) from the volume of voxels with positive activity (new and enlarging). See Fig. 2 for an example of the classification of lesion changes.

We performed a quality check on the output for all intervals (i.e., visual inspection of errors in the registration to halfway space and artifacts in the subtraction image such as described by Duan et al. (2008)). An interval of a subject was excluded if, due to incomplete brain coverage, the anatomical location of a lesion was inside the field of view in one visit but not in the other. Furthermore, an interval of a subject was included only if all corresponding lesion and atrophy measurements were available.

### 2.3 Atrophy measures

Yearly global and central atrophy were measured by estimating the percentage brain volume change (PBVC) and percentage ventricular volume change (PVVC) respectively, by using SIENA and its extension VIENA (Vrenken et al., 2014) both part of FSL (version 6.0.3). A more negative and more positive value are indicative of more atrophy for PBVC and PVVC respectively. The PBVC and PVVC for intervals longer than 1 year were calculated by:

$${\text{PBVC}}_{years\;i\;through\;N}=\left(\left(1+\frac{{\text{PBVC}}_{i}}{100}\right)∙\left(1+\frac{{\text{PBVC}}_{i+1}}{100}\right)∙\ldots ∙\left(1+\frac{{\text{PBVC}}_{N}}{100}\right)-1\right)∙100$$

$${\text{PVVC}}_{years\;i\;through\;N}=\left(\left(1+\frac{{\text{PVVC}}_{i}}{100}\right)∙\left(1+\frac{{\text{PVVC}}_{i+1}}{100}\right)∙\ldots ∙\left(1+\frac{{\text{PVVC}}_{N}}{100}\right)-1\right)∙100$$

### 2.4 Validation procedure and statistical analyses

Statistical analyses were performed with IBM SPSS Statistics (version 28). For our validation purposes, month 60 was used as the reference instead of the baseline visit because of the design of the REFLEXION trial, where patients are recruited just after a first attack. For this reason, we expect the early treatment group to suffer from pseudo-atrophy, i.e., shifts in fluid caused by the initiation of anti-inflammatory medication leading to a reduction in brain volume without actual cell loss in the first 6 months to 1 year (De Stefano et al., 2014; Zivadinov et al., 2008), which might disturb the measurements in the first year of the study.

The performance of the semi-automated method was evaluated for three different situations.

1. The performance for the intended use, i.e., yearly lesion volume change quantification. All five yearly intervals were pooled to assess the accuracy and the two-yearly interval month (M)36-M60 was used to assess the reproducibility and accuracy.
2. The reproducibility and accuracy was assessed for “longer intervals”, namely: M24-M60 (3 years), M12-M60 (4 years), and BSLN-M60 (5 years).
3. The relation of several factors with the method's performance, namely:
  a Global atrophy (PBVC) and central atrophy (PVVC).
  b Lesion volume, defined as the average lesion volume of two manual lesion volume assessments and lesion volume change, defined as the numerical difference between two manual lesion volume assessments.

For atrophy, the two-yearly (M36-M60) and longer intervals (M24-M60, M12-M60, and BSLN-M60) were assessed. For lesion volume (change) only the M36-M60 interval was assessed to minimize the influence of other factors such as (pseudo)atrophy.

The reproducibility of the semi-automated method was evaluated through a transitivity error analysis. This was performed by calculating the intraclass correlation coefficient for the absolute agreement (ICC) between an indirect multi-step TLVC and direct one-step TLVC. The one-step TLVC was calculated directly from the subtracted images of visit 1 and visit N. The indirect multi-step TLVC was determined by:

$${\text{TLVC}}_{multi-step}=\sum_{year=1}^{N}{\text{TLVC}}_{year}$$

Additionally, the SD of the difference between the multi-step and one-step TLVC was calculated. To assess the relation of the different factors (PBVC, PVVC, and lesion volume (change)) with the reproducibility of the method, the absolute difference between the multi-step and one-step TLVC was calculated as well. Then the Spearman's correlation coefficients between this absolute difference and atrophy/lesion volume (change) were calculated.

The accuracy was assessed by calculating the ICC between the one-step TLVC and the manually measured lesion volume change (LVC). The mean difference between the one-step TLVC and manual LVC was also reported together with the results of a paired *t*-test. To assess the relation of the different factors with the accuracy of the method, the Spearman's correlation coefficients (r_s_) between this difference and atrophy/lesion volume (change) were calculated. An alpha of 0.05 was used as the cut-off for significance for all analyses.

### 2.5 Data and code availability statement

Any requests for data by qualified scientific and medical researchers for legitimate research purposes will be subject to the Data Sharing Policy of the healthcare business of Merck KGaA, Darmstadt, Germany. All requests should be submitted in writing to the data sharing portal for the healthcare business of Merck KGaA, Darmstadt, Germany https://www.emdgroup.com/en/research/our-approach-to-research-and-development/healthcare/clinical-trials/commitment-responsible-data-sharing.html. When the healthcare business of Merck KGaA has a co-research, co-development, or co-marketing or co-promotion agreement, or when the product has been out-licensed, the responsibility for disclosure might be dependent on the agreement between parties. Under these circumstances, the healthcare business of Merck KGaA will endeavor to gain agreement to share data in response to requests. The source code to perform the different steps as described in section 2.2 is available at the following link: https://gitlab.com/sbig/lesionchange.

## 3 Results

### 3.1 Performance during intended use

**Fig. 3:**
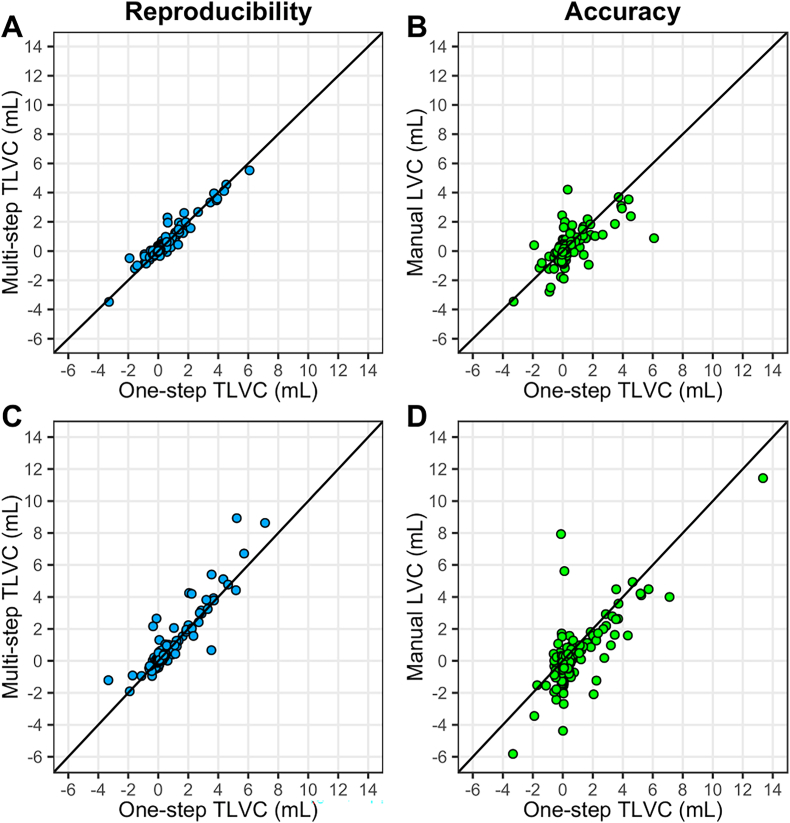
Scatterplots showing the agreement between the one-step and multi-step measurements of total lesion volume change (TLVC; in mL). The reproducibility in the two-yearly interval month 36-month 60 (M36-M60; panel A) and four-yearly interval month 12-month 60 (M12-M60; panel C) is shown. The agreement between the one-step semi-automated measurement and manually measured lesion volume change (LVC; in mL) as an indication of accuracy in the two-yearly interval M36-M60 (panel B) and four-yearly interval M12-M60 (panel D) is also shown. Identity lines, reflecting perfect agreement, are shown for reference.

The reproducibility, assessed as ICC between the direct one-step and indirect multi-step TLVC for the two-yearly M36-M60 interval was excellent with ICC = 0.96, 95% CI [0.95, 0.97]. See also Fig. 3 panel A for a scatterplot. The SD of the difference between one-step and multi-step TLVC was 0.25 mL.

The accuracy, assessed as ICC between yearly semi-automatically measured one-step TLVC and manually measured LVC was good with ICC = 0.86, 95% CI [0.85, 0.88]. The absolute agreement was moderate for the two-yearly M36-M60 interval (ICC = 0.67, 95% CI [0.59, 0.74]). See also Fig. 3 panel B for a scatterplot. The mean difference of 0.11 mL between one-step TLVC and manual LVC was the same for both intervals and significantly different from zero (pooled yearly intervals: p < 0.001; M36-M60: p = 0.019).

### 3.2 Limits of performance

The semi-automated method showed good to excellent reproducibility, with an ICC range of 0.90–0.94 between the one-step and multi-step TLVC for a follow-up period from 3 up to 5 years. The SD of the difference between one-step and multi-step TLVC ranged from 0.55 to 0.86 mL. See Table 2 for more detailed results and Fig. 3 panel C for a scatterplot displaying the reproducibility of M12-M60.

**Table 2:** Assessment of reproducibility and accuracy for longer intervals.

Years	Interval	Reproducibility		Accuracy	
		ICC [95% CI] between one-step and multi-step TLVC	SD of transitivity error	ICC [95% CI] between one-step TLVC and manual LVC	Mean difference between one-step TLVC and manual LVC (p-value)
3	M24-M60	0.90 [0.87, 0.92]	0.55 mL	0.77 [0.70, 0.82]	**0.13 mL (0.017)**
4	M12-M60	0.94 [0.92, 0.95]	0.58 mL	0.75 [0.65, 0.81]	**0.37 mL (< 0.001)**
5	BSLN-M60	0.93 [0.90, 0.95]	0.86 mL	0.85 [0.80, 0.89]	**0.34 mL (< 0.001)**

Bold font indicates statistical significance (p < 0.05). BSLN = baseline, CI = confidence interval, ICC = intraclass correlation coefficient, LVC = lesion volume change, M = month, SD = standard deviation, TLVC = total lesion volume change.

The accuracy was good, with the ICC between the one-step TLVC and manual LVC ranging from 0.75 to 0.85. The mean difference was significantly different from zero and ranged from 0.13 to 0.37 mL. See Table 2 for more detailed results and Fig. 3 panel D for a scatterplot displaying the accuracy of M12-M60.

### 3.3 Relation between atrophy and performance

**Fig. 4:**
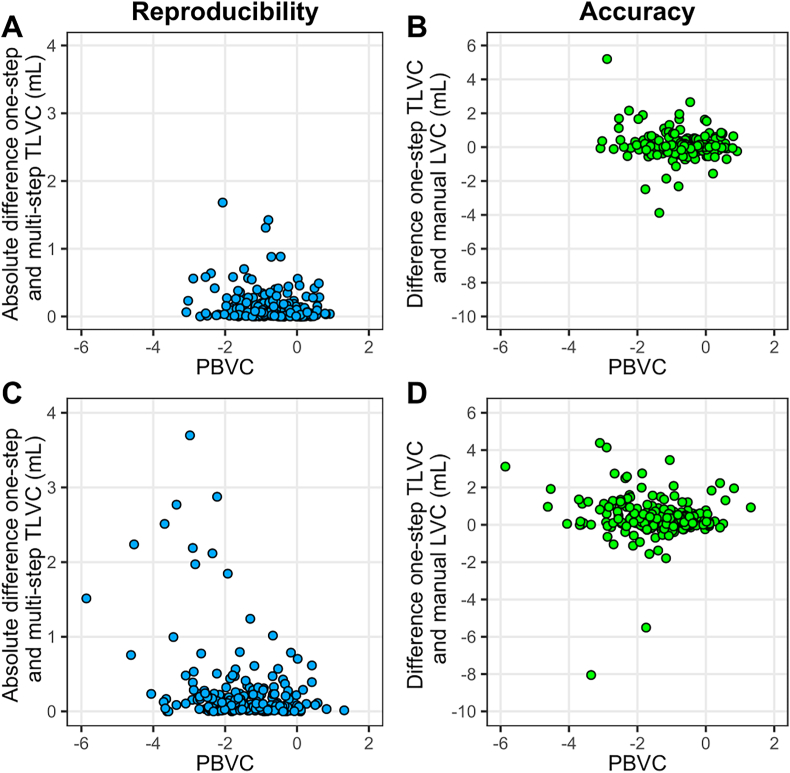
Scatterplots showing the relation between reproducibility/accuracy and global atrophy. Panel A shows the scatterplot for the absolute difference between the one-step and multi-step measurements of total lesion volume change (TLVC; in mL) as an indication of reproducibility and its relation with percentage brain volume change (PBVC; global atrophy) for the two-yearly interval month 36-month 60 (M36-M60). Panel C shows this for the four-yearly interval month 12-month 60 (M12-M60). Panel B shows the scatterplot for the difference between the one-step TLVC and manual lesion volume change (LVC; in mL) as an indication of accuracy and its relation with PBVC for the two-yearly interval M36-M60. Panel D shows this for the four-yearly interval M12-M60.

Both global (PBVC) and central (PVVC) atrophy were significantly correlated with the reproducibility. More atrophy was associated with a larger absolute difference between one-step and multi-step TLVC (PBVC: r_s_ range = −0.19 to −0.28; PVVC: r_s_ range = 0.22 to 0.34). There was generally no significant correlation between global/central atrophy and the accuracy. See Table 3 for more details and Fig. 4 for the scatterplots of M36-M60 (panel A and B) and M12-M60 (panel C and D).

**Table 3:** Correlations to assess the relation between global (PBVC)/central atrophy (PVVC) and the reproducibility/accuracy.

Years	Interval	PBVC		PVVC	
		Absolute deviation one-step and multi-step TLVC	Deviation one-step TLVC and manual LVC	Absolute deviation one-step and multi-step TLVC	Deviation one-step TLVC and manual LVC
2	M36-M60	**r**_**s**_ = −**0.19**	r_s_ = −0.04	**r**_**s**_**= 0.25**	r_s_ = 0.04
		**p = 0.004**	p = 0.522	**p < 0.001**	p = 0.547
3	M24-M60	**r**_**s**_**=** −**0.28**	r_s_ = −0.01	**r**_**s**_**= 0.32**	r_s_ = −0.01
		**p < 0.001**	p = 0.936	**p < 0.001**	p = 0.941
4	M12-M60	**r**_**s**_**=** −**0.28**	r_s_ = −0.12	**r**_**s**_**= 0.34**	r_s_ = 0.13
		**p < 0.001**	p = 0.096	**p < 0.001**	p = 0.072
5	BSLN-M60	**r**_**s**_**=** −**0.20**	**r**_**s**_**=** −**0.14**	**r**_**s**_**= 0.22**	r_s_ = 0.14
		**p = 0.005**	**p = 0.049**	**p = 0.002**	p = 0.050

Bold font indicates statistical significance (p < 0.05). BSLN = baseline, LVC = lesion volume change, M = month, PBVC = percentage brain volume change, PVVC = percentage ventricular volume change, r_s_ = Spearman's correlation coefficient, TLVC = total lesion volume change.

### 3.4 Relation between manually measured lesion volume (change) and performance

**Fig. 5:**
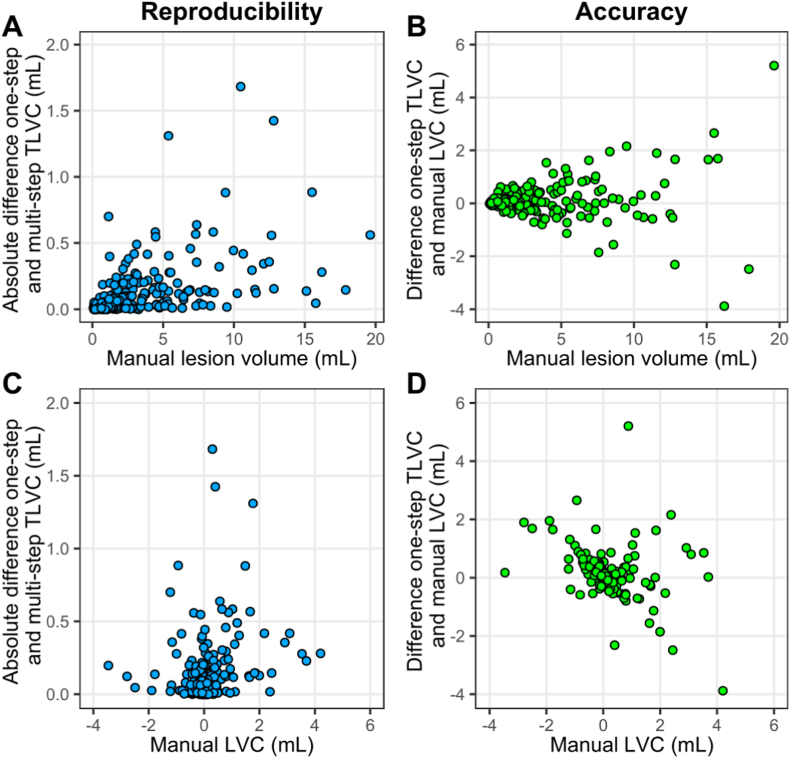
Scatterplots showing the relation between the reproducibility/accuracy and manual lesion volume/lesion volume change in the two yearly interval month 36-month 60. Panel A shows the scatterplot for the absolute difference between the one-step and multi-step measurements of total lesion volume change (TLVC; in mL) as an indication of reproducibility and its relation with the average manual lesion volume. Panel C shows the relation between reproducibility and manual lesion volume change (LVC; in mL). Panel B shows the scatterplot for the difference between the one-step TLVC and manual lesion volume change (LVC; in mL) as an indication of accuracy and its relation with manual lesion volume. Panel D shows the relation between accuracy and manual LVC.

A higher manually measured (average) lesion volume was related to a lower reproducibility of the semi-automated method as indicated by a significant positive correlation between lesion volume and the absolute difference between the one-step and multi-step TLVC (r_s_ = 0.62, p < 0.001, see Table 4). A higher manual LVC was related to a lower performance of the method as the manual LVC was significantly positively correlated with the absolute difference between one-step and multi-step TLVC (reproducibility: r_s_ = 0.22, p < 0.001) and a higher manual LVC was significantly negatively correlated with the difference between one-step TLVC and manual LVC (accuracy: r_s_ = −0.52, p < 0.001). The scatterplot in Fig. 5 panel D shows that the latter result indicates that for a positive manual LVC the one-step TLVC is systematically lower than the manual LVC and vice versa for a negative manual LVC.

**Table 4:** Correlations to assess the relation between manual lesion volume/manual lesion volume change and reproducibility/accuracy.

Manual lesion volume		Manual LVC	
Absolute deviation one-step and multi-step TLVC	Deviation one-step TLVC and manual LVC	Absolute deviation one-step and multi-step TLVC	Deviation one-step TLVC and manual LVC
**r**_**s**_**= 0.62**	r_s_ = 0.08	**r**_**s**_**= 0.22**	**r**_**s**_**=** −**0.52**
**p < 0.001**	p = 0.214	**p < 0.001**	**p < 0.001**

Bold font indicates statistical significance (p < 0.05). LVC = lesion volume change, r_s_ = Spearman's correlation coefficient, TLVC = total lesion volume change.

## 4 Discussion

The results of the current study indicate that the proposed subtraction method is a valid and robust approach to semi-automatically quantify lesion volume changes over time in (early) multiple sclerosis. The reproducibility was excellent and the overall accuracy was good for follow-up periods between 1 and 5 years. More atrophy and higher lesion volume have a limited negative impact on the reproducibility and higher lesion volume change on both the reproducibility and accuracy.

The semi-automated method provides an insight into the lesion volume changes using serial PD-weighted MRI scans as input. We could not assess the scan-rescan reproducibility because only one set of scans was performed per visit for the REFLEX/REFLEXION studies. For this reason, we chose to assess the reproducibility by measuring the absolute agreement between the direct one-step and indirect multi-step measurement of TLVC. A similar approach of performing a transitivity error analysis as part of a validation study has been performed in other studies as well (e.g., Smith et al., 2001; Smith et al., 2002). The reproducibility of the current semi-automated method was excellent with ICC values ranging from 0.90 to 0.96 across all intervals, which indicates that the method is very precise for follow-up periods between 1 and 5 years.

There was, overall, good agreement between the TLVC as quantified by the one-step semi-automated method and the LVC as calculated by numerically subtracting two manually measured lesion volume assessments. The ICC ranged from 0.67 to 0.86. Hence, the measurements resulting from these two different approaches were comparable but not directly interchangeable. A potential explanation for these differences is that the manually measured lesion volume change requires two separate measurements of a patient, which introduces two occasions where measurement errors (e.g., missed voxels) could occur. In the proposed semi-automated method, the longitudinal aspect is taken into account by implementing a registration to halfway space and the use of subtraction images, which reduces the effect of repositioning errors, and active disease is enhanced against the background (Moraal et al., 2009). Conversely, the semi-automated method could fail to quantify small subtle changes if a voxel does not reach the threshold of Z > |1.5| after all the (pre)processing steps or if a voxel falls outside the registered manual lesion masks which are used as a reference.

The median lesion volume at the start of the REFLEXION study (month 24) was 1.9 mL. The estimated yearly change in lesion volume is about 10% in untreated patients (Paty et al., 1994). Considering this, the estimated yearly change in lesion volume would be 0.19 mL. The SD of the difference between the one-step and multi-step TLVC reflecting the reproducibility, and especially the mean difference between the one-step TLVC and manual LVC reflecting the accuracy, fall below the estimated yearly lesion volume change extrapolated to the corresponding intervals listed in Table 2. This indicates that the error in the performance of the semi-automated method is not as high as the actual lesion volume change that it aims to detect, which provides further support for the reliability of the method.

Both global and central atrophy seemed to have a limited negative impact on the reproducibility of the method only, that is, the higher the atrophy rate, the lower the reproducibility. A potential explanation for this could be that faster atrophy between visits might increase misregistration. However, the Spearman correlation coefficients were generally small and if we look at Fig. 4 panels A and C this relationship does not seem to be very prominent and clearly interpretable. Given that the annual brain volume loss is estimated to be between 0.5 and 1.35% in patients with MS (De Stefano et al., 2014) most data points around this atrophy value for the reproducibility fall within the 0.19 mL estimated yearly lesion volume change (note that panels A and C refer to a two-yearly and four-yearly interval, respectively). The patient population for the current study concerns patients with early MS. How well the method performs in more advanced stages of the disease and progressive disease types of MS needs to be investigated in future studies.

A higher (manually measured) average lesion volume seemed to have a negative impact on the reproducibility. A higher lesion volume change was related to both lower reproducibility and accuracy. Especially the regression coefficients of the relation between lesion volume and reproducibility, and the relation between lesion volume change and accuracy, were strong. A positive change in the lesion volume seemed to be related to an underestimation in the one-step TLVC as compared to the manual LVC, and a negative change to an overestimation as shown in Fig. 5 panel D.

In the current study, we assessed the validity of the semi-automated method by using the TLVC metric, while this method can also, more specifically, provide an insight into the negative and positive lesion volume changes and, even more specifically, the different lesion categories (i.e., disappearing, shrinking, new, and enlarging). Because of the approach we used to validate the method, we could only do this for the TLVC and not the different categories. However, since the TLVC is a combined measure of all possible lesion changes and would not be reliable if the underlying categories were measured incorrectly, this can be considered as indirect evidence for the validity of the different categories of lesion change. It is an advantage of the current method that it can provide quantitative information about the whole spectrum of changes, both positive (new and enlarging) and negative (disappearing and shrinking), as the latter is an underexposed topic and metric, because in clinical practice new (breakthrough) disease activity is considered as very important (Kaunzner and Gauthier, 2017).

A disadvantage of the current method is that it requires pre-existing lesion masks as a reference region, and these are often not available as this is very labor-intensive. However, this method could be combined with existing (semi-)automated methods such as the method by Storelli et al. (2016) which can semi-automatically segment lesions on PD-weighted images. A comprehensive review of the developments in methods concerning the segmentation of lesions and lesion dynamics is provided by Lladó et al. (2012).

Currently there is a tendency to move towards (3D) FLAIR imaging because of the consensus recommendations on the use of MRI in patients with MS (Wattjes et al., 2021). However, PD/T2 dual-echo imaging is still acquired and has been used in many large clinical trials. In order to analyze legacy data, methods that are able to use these more “old fashioned” conventional MR images as input are needed. Historical datasets such as the REFLEX/REFLEXION studies can provide a lot of useful insights, given that there was a placebo group which would nowadays not be ethically feasible given the proven effectiveness of disease-modifying treatments.

In conclusion, the current proposed semi-automated method to quantify lesion volume changes has excellent reproducibility and overall good accuracy. When applying this method, the amount of atrophy and especially lesion volume (change) should be taken into account, as an increase in these variables might affect the quality of the results. Overall, the semi-automated subtraction method can reliably quantify lesion volume changes over time in (early) multiple sclerosis for follow-up periods up to 5 years.

## Declaration of competing interest

The authors declare the following financial interests/personal relationships which may be considered as potential competing interests:

**RMM** has received research support from Merck. **IB** has received research support from Merck, Novartis, Teva, and the Dutch MS Research Foundation. **BMJU** reports research support and/or consultancy fees from Biogen Idec, Genzyme, Merck, Novartis, Roche, Teva, and Immunic Therapeutics. **FB** is a steering committee or Data Safety Monitoring Board member for Biogen, Merck, ATRI/ACTC and Prothena. Consultant for Roche, Celltrion, Rewind Therapeutics, Merck, IXICO, Jansen, Combinostics. Research agreements with Merck, Biogen, GE Healthcare, Roche. Co-founder and shareholder of Queen Square Analytics LTD. **HV** has received research support from Merck, Novartis, Pfizer, and Teva, consulting fees from Merck, and speaker honoraria from Novartis; all funds were paid to his institution. **SS, ASM, AV, RAvS,** and **JPAK** report no disclosures.

## Data Availability

Data will be made available on request and the code is publicly available (please see section 2.5 for more details).
